# From glitter to gold: recommendations for effective dashboards from design through sustainment

**DOI:** 10.1186/s13012-025-01430-x

**Published:** 2025-04-22

**Authors:** Fernanda S. Rossi, Meredith C. B. Adams, Gregory Aarons, Mark P. McGovern

**Affiliations:** 1 Stanford Center for Dissemination and Implementation (CDI), Department of Psychiatry and Behavioral Sciences, Stanford University School of Medicine, Palo Alto, CA USA; 2 Department of Anesthesiology, Artificial Intelligence, Translational Neuroscience and Public Health Sciences, Wake Forest University School of Medicine, Winston-Salem, NC USA; 3 Department of Psychiatry and ACTRI Dissemination and Implementation Science Center, University of California San Diego, La Jolla, San Diego, CA USA; 4 Stanford Center for Dissemination and Implementation (CDI), Department of Psychiatry & Behavioral Sciences, Stanford University School of Medicine, 1070 Arastradero Road, Suite 371, Palo Alto, CA 94304 USA

**Keywords:** Dashboards, Human-centered design, Implementation science, Dashboard use, EPIS

## Abstract

**Background:**

Dashboards—tools that compile and summarize key performance data—have become increasingly utilized for supporting data organization and decision-making processes across various fields, such as business, economics, healthcare, and policy. The dashboard’s impact is dependent on its use by the individuals for whom it was designed. Yet, few studies measure dashboard use, and of those that do, their utility is limited. When dashboards go unused, they provide little value and impact. We argue that successful and long-term use of dashboards can be achieved using human-centered design and implementation science methods.

**Main body:**

In this article, we describe the characteristics of dashboards and provide examples of existing dashboards. We discuss the common pitfalls of dashboards that result in their limited use. Next, we proffer how human-centered design and implementation science can improve dashboard relevance. We provide eight recommendations from across the design to the sustainment phase. To guide dashboard developers and implementers, we organize our recommendations using the Exploration, Preparation, Implementation, and Sustainment (EPIS) Framework. Lastly, we raise several cautions when using human-centered design and implementation science methods in dashboard development and implementation.

**Conclusion:**

There is a need for more effective, sustainable, and impactful dashboards. We suggest that incorporating human-centered design and implementation science methods can facilitate achieving this goal.

Contributions to Literature

- Human-centered design and implementation science methods may be critical for developing more sustainable and impactful dashboards that effectively promote evidence-based care practices in the community.
- We highlight challenges dashboard developers and implementers often encounter that limit dashboard use. We offer recommendations and examples of how to successfully address these challenges, incorporating human-centered design and implementation science methods across the dashboard design to sustainment process to improve dashboard use.
- Though there are many benefits to incorporating human-centered design and implementation science methods to dashboard development and implementation, we describe potential cautions to this approach.

## Background

Dashboards—tools that compile and summarize key performance data—have become increasingly popular for supporting data organization and decision-making processes across various fields, such as business, economics, healthcare, and policy [1]. Dashboard developers and implementers often spend significant time designing and implementing dashboards. The dashboard’s impact is dependent on its use by the individuals it was designed for. Yet, two systematic reviews of dashboards in health care settings demonstrate that most studies fail to measure dashboard use [1, 2]. Only one study examined dashboard use and indicated that few individuals (28%) used the dashboard at least once [3]. When dashboards go unused, they provide little value and impact.

We argue that successful and long-term use of dashboards can be achieved using human-centered design and implementation science methods. We, first, describe the characteristics of dashboards and provide examples of existing dashboards. Second, we describe the common pitfalls of dashboards that result in their limited use. Third, we introduce the fields of human-centered design and implementation science, focusing on aspects of each field that can inform dashboard use. We list eight recommendations across the dashboard design to sustainment process to improve dashboard use, incorporating elements from human-centered design and implementation science. Finally, we describe the potential cautions to adopting human-centered design and implementation science methods in dashboard development and implementation.

### Dashboard characteristics

Dashboards are specialized tools intended to support data aggregation and reporting using data visualizations to facilitate the monitoring, interpretation, and improvement of key metrics [4]. By compiling meaningful and often complex information, dashboards can serve different purposes. Some dashboards display information on *what has been done* in a particular setting such as by helping organizations or end users coordinate data from multiple sources, detect data trends, observe the impact of a practice or policy, monitor performance or quality metrics, identify population characteristics, track benchmark achievements, and/or manage workflow processes [1, 4–6]. Other dashboards provide information on *what to do* such as by helping organizations or end users make data-driven decisions, form normative data and peer comparisons, prompt adherence to an evidence-based practice or policy, and/or better utilize available resources [1, 4–7]. Dashboards display various characteristics that help them communicate information.

To communicate information, dashboards often incorporate color coding, tables, graphs, and/or symbols [4, 8]. Information can be displayed using diverse platforms. Most dashboards rely on online platforms, such as websites, the electronic medical record, or software applications, that automate information and allow end users to interact with dashboard content [4]. Digital dashboards offer a unique opportunity to compile real-time data and provide immediate information [1, 7]. However, it is also possible for dashboards to compile data electronically and communicate information via more simplified and less interactive platforms, such as newsletters, flyers, and email [4]. Dashboards generally offer an advantage over more traditional methods of providing information that may require significant time to manually compile, summarize, and share data [1, 7].

### Examples of dashboards

Dashboards can be found across a wide range of settings and fields (e.g., business, public health, economics, healthcare, policy). They can also operate at different societal levels, from individual, clinic, organizational, and community levels, to serving large populations and nations. Public health surveillance dashboards, for instance, monitor and predict health-related trends or disease outbreaks in particular communities or nations [6, 7, 9, 10]. Policy dashboards, on the contrary, may aggregate data that help inform understanding of the impact of local or national policies or laws [6, 11].

Dashboards have become especially prevalent in healthcare settings. Healthcare organizations acquire significant information regarding patients, quality of care, and administrative processes, often across fragmented systems. Dashboards are one method for integrating and summarizing fragmented healthcare data in a meaningful and interpretable manner [8]. Within the healthcare context, dashboards are often characterized as clinical dashboards, administrative dashboards, or both [1, 4]. Clinical dashboards capture clinical data that can help with tasks such as directing patient care, coordinating care, or identifying patients at risk of an adverse event or in need of intervention [4]. Administrative dashboards, on the other hand, capture administrative information to help with tasks such as comparing providers on performance metrics, tracking healthcare utilization, and managing resources [4]. Some dashboards capture both clinical and administrative data. Clinical and/or administrative dashboards have been designed to address numerous problems in healthcare settings. For example, Tan and colleagues created a dashboard that provides clinical recommendations for high risk patients likely to benefit from primary palliative care [12]. Another dashboard by Sheen and colleagues was designed to monitor and improve glycemic management among hospitalized adults [13].

### Common challenges with dashboards

Despite the potential advantages of dashboards, we have noted, via the literature and in our own research experiences, the frequent challenges or pitfalls of dashboards that lead to their limited use [7, 14–16]. We describe each of these challenges below, which are commonly observed across all types of dashboards.

#### #1: The dashboard lacks value to end users

Dashboard developers and implementers may design dashboards that display information they believe are critical for achieving the dashboard’s predetermined goals. However, such information may not be equally important or informative to end users. That is, the value proposition for end users of the dashboard is often not assessed or understood [17]. Dashboards that lack value can appear in different forms. For instance, a dashboard may display content that end users find unhelpful or content that lacks actionable information with end users unable to determine appropriate next steps informed by the dashboard data. When a dashboard lacks value to end users, they are less likely to adopt and use the dashboard. As one example, author M.P.M. and colleagues are conducting a study aimed at increasing access to medications for opioid use disorder (MOUD) in specialty addiction programs in Washington State. This study uses dashboards to provide clinic leaders and staff with performance data as well as data on how each program compared to other programs [18, 19]. An initial version of the dashboard at the start of the study included information the researchers thought would be critical for helping programs increase access to MOUD. However, during the study, it became apparent that clinic leaders and staff rarely logged in to the dashboard as they did not find it valuable to their practice.

#### #2: The dashboard relies on incomplete, outdated, inaccurate, or biased data

Dashboards are only as accurate as the data they use [20]. Thus, if data are incomplete, outdated, inaccurate, or biased, any data summaries provided by the dashboard to end users will be incorrect or irrelevant, consequently, decreasing the dashboard’s clinical value and use. The Healing Communities Study dashboards, which provide communities with opioid-related data (described in more detail below), showcase the frequent lag dashboards have in acquiring data and the efforts needed to decrease that lag [7]. Incomplete, outdated, inaccurate, or biased dashboards can have long-lasting negative consequences, such as by providing false or outdated conclusions or excluding certain minoritized groups and perpetuating health inequities. For instance, a dashboard that relies on data from mostly White individuals will likely provide biased information that poorly reflects minoritized groups, such as Black and Latinx individuals.

#### #3: The dashboard results in unintended consequences

Some dashboards are designed to provide performance information or facilitate peer comparisons. Although such information is meant to encourage behavior change, it may have an unintended negative impact on end users [21]. For instance, an end user that receives negative performance information may develop negative self-perceptions. This can then lead to feelings of discouragement and resentment about their work as well as negative attitudes about the organization. As such, the dashboard may inadvertently encourage the end user to ignore the dashboard to avoid receiving negative feedback. Dashboards can also generate unintended consequences by perpetuating stigma. For example, the U.S. Department of Justice’s Crime in the United States dashboard displays data on arrests according to race and type of crime, which while helpful to address crime, can stigmatize certain races [22].

#### #4: The dashboard is difficult to sustain

Difficulties with dashboard sustainment may be due to various factors, including if the dashboard is poorly integrated with end users’ workflow processes, is difficult to understand or access, or requires significant expertise or resources to maintain [16]. Some dashboards may be costly due to software licensing costs, and some may require an administrative or data management team to manage its functioning. Dashboards may also depend on complex data acquisition and sharing processes. These processes may require regular security updates to ensure data protection. Dashboard developers and implementers, within the context of a research study, for example, may be able to provide the necessary administrative and funding support to help with dashboard adoption. However, when the study ends and such administrative and funding supports are no longer available, it can be challenging to sustain long-term dashboard use. The Healing Communities Study dashboards provide an example of the important need to address sustainability barriers, like software costs, complex acquisition and data sharing processes, and complex system management [7].

### Addressing challenges using human-centered design and implementation science methods to enhance dashboard use

To address the challenges with dashboards, dashboard developers and implementers can incorporate human-centered design and implementation science methods to enhance dashboard use. Human-centered design focuses on integrating innovations into the real-world [23] by bringing end users and developers together to co-develop an innovation using an iterative and creative process [23, 24]. It emphasizes the importance of the end user perspective in product development for improving utility, uptake, sustainability, and effectiveness of the product [24].

Like human-centered design, implementation science focuses on translating evidence-based practices, innovations, and policies from research to practice. However, it uses scientific methods to examine and enhance the implementation process by addressing and identifying implementation challenges [25]. Implementation science places value on the context in which implementation takes place, considering its various levels from the inner context (e.g., individual, organizational) to the outer context (e.g., system, policy) [23, 25–27]. Context helps inform barriers and facilitators to implementation (i.e., factors that hinder or enhance implementation), effective implementation strategies (i.e., methods used to deliver the innovation), and appropriate implementation outcomes that help measure successful implementation [23, 25]. As a result of implementation science, there have been significant strides in bringing evidence-based treatments to routine clinical care—one example being the twofold increase in uptake of medications for opioid use disorder [28].

Only recently have we begun to consider the synergistic effects of human-centered design and implementation science in the implementation of health innovations. Both fields are complementary, with some overlapping goals or concepts yet distinct contributions that can help strengthen our ability to translate evidence-based innovations into real world applications [23]. Though both fields, for example, seek to improve innovation uptake, human-centered design does so by gathering iterative feedback on the innovation from end users while implementation science focuses on the challenges to implementation across differing contexts [23–25]. Both sets of information are important and together can enhance innovation uptake. The incorporation of human-centered design and implementation science methods to dashboard development and implementation is innovative [29]. Human-centered design methods may be especially helpful in bolstering dashboard development given its emphasis on end user perspectives during product development. Implementation science methods, on the other hand, may be especially helpful in bolstering dashboard implementation given its emphasis on addressing and identifying implementation challenges. We believe that this combined human-centered design and implementation science approach can help address the many challenges with dashboards and lead to highly effective and sustainable dashboards. We list below eight recommendations for how dashboard developers and implementers can incorporate human-centered design and implementation science methods to achieve sustained dashboard use. Our recommendations can be universally applied to all types of dashboards.

### Using the Exploration, Preparation, Implementation, and Sustainment (EPIS) framework to guide dashboard development and implementation

To help guide dashboard developers and implementers in developing and implementing their dashboards, we organize our recommendations using the Exploration, Preparation, Implementation, and Sustainment** (**EPIS) Framework, a widely-known framework in implementation science that outlines and describes four phases in the development and implementation process—exploration, preparation, implementation, and sustainment [26]. Although the process begins in the exploration phase, it is important to think about sustainment from the beginning so that all activities lead to sustained impact. The exploration phase focuses on exploring the emergent or existing needs of individuals, communities, or systems and identifying the most appropriate innovations to address those needs. The preparation phase focuses on identifying barriers and facilitators to implementation of the innovation and developing a comprehensive plan that addresses those barriers and capitalizes on the facilitators. During the implementation phase, the innovation is introduced to the system or organization. In the sustainment phase, the innovation continues to be delivered or used long-term via ongoing structures, supports, and processes. All phases consider the inner (e.g., patient characteristics) and outer contexts (e.g., organizational characteristics) as well as bridging (e.g., community-academic partnerships) and innovation factors (e.g., characteristics of the innovation) that may influence implementation [26, 27].

**Fig. 1:**
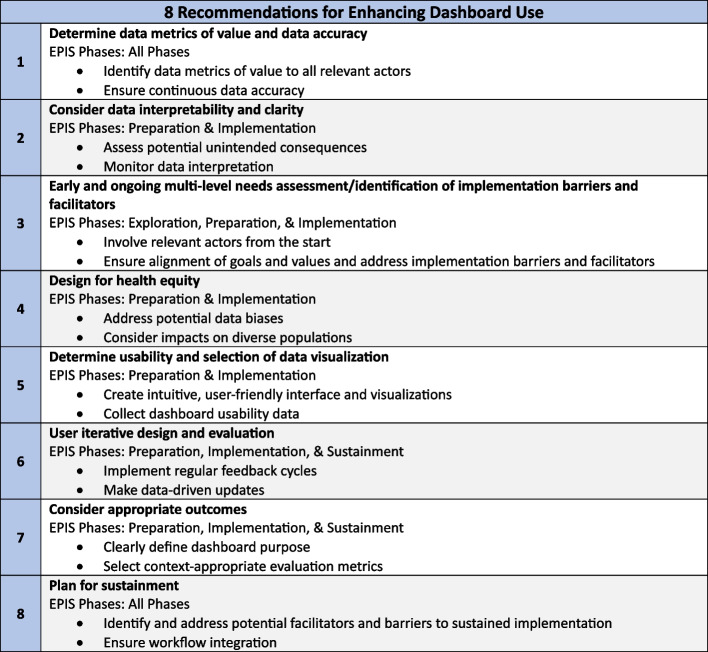
Summary of eight recommendations to enhance dashboard use. Note. EPIS is the Exploration, Preparation, Implementation, and Sustainment Framework

**Fig. 2:**
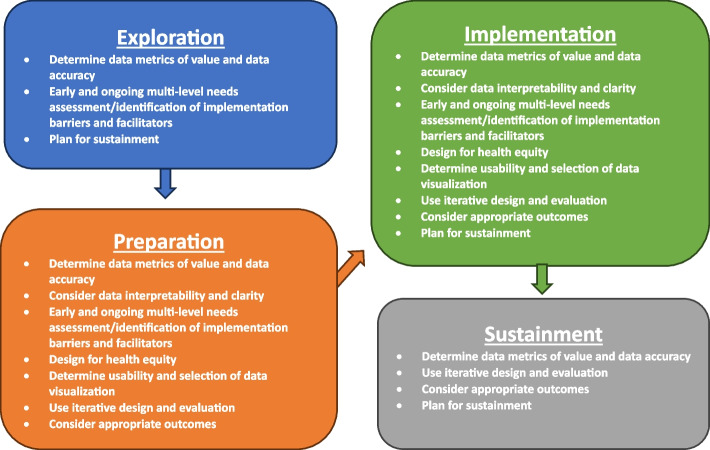
Recommendations for enhancing dashboard use organized by EPIS phase. Note. EPIS is the Exploration, Preparation, Implementation, and Sustainment Framework

The EPIS Framework provides an excellent roadmap for dashboard developers and implementers to implement effective and sustainable dashboards. Thus, we label each of our recommendations below according to the EPIS phase in which the recommendation should be enacted or considered. Many recommendations are relevant across multiple phases due to their iterative and ongoing nature. Our recommendations suggest that use of human-centered design methods is especially important during the EPIS exploration and preparation phases while implementation science methods are especially important during the implementation and sustainment phases. Figure 1 provides a summary of recommendations, and Fig. 2 shows recommendations organized by EPIS phase.

### Recommendations for enhancing dashboard use

#### #1: Determine data metrics of value and data accuracy (EPIS Phases: Exploration, Preparation, Implementation, and Sustainment)

As informed by both human-centered design and implementation science, it is critical for dashboard developers and implementers to identify dashboard data metrics (i.e., information displayed on the dashboard) valuable to relevant actors (e.g., what kind of data do they find important and helpful; do they trust the data; are the data presented in ways that align with their values, such as the preservation of confidentiality). Relevant actors include end users and other individuals who may not directly interact with the dashboard but are impacted by it or help maintain its use, such as organizational leaders [14]. Dashboards with data metrics valuable to all relevant actors are more likely to be adopted and sustained. To identify which data metrics are valuable to end users, for example, dashboard developers and implementers must identify the dashboard end user population and select representatives from that population who would be willing to provide feedback on the dashboard’s data metrics, such as through qualitative interviews or surveys [14, 30]. Interview or survey questions may be informed by the Consolidated Framework for Implementation Research (CFIR), which helps guide systematic assessment of barriers and facilitators to implementation of an innovation and necessary tailoring or adaptions to that innovation [31, 32]. End users and other relevant actors can differ according to dashboard purpose and type. End users of clinical dashboards may include health providers while end users of administrative dashboards may include non-clinical staff. For both types of dashboards, other relevant actors may include clinical management. It is critical to acquire feedback from all relevant actors during the EPIS exploration, preparation, implementation, and sustainment phases as the data metric needs and values of relevant actors may change.

For data metrics to be valuable to end users and other actors, dashboard developers and implementers must also ensure dashboard data accuracy. Dashboards must continuously display the most recent and accurate data to be useful and promote effective decision-making [14]. Thus, dashboard developers and implementers must carefully consider, particularly during the preparation phase, how data will be acquired, data accuracy, and the timing of data acquisition [15]. Dashboard developers and implementers must then use this information to address any potential data inaccuracies and lags in data acquisition [15].

#### #2: Consider data interpretability and clarity (EPIS Phases: Preparation and Implementation)

It is critical that dashboard developers and implementers consider the dashboard’s data interpretability and clarity and ensure that end users are accurately interpreting dashboard data and communications. This is consistent with human-centered design methods, which place end users at the center of the design process [24]. Accurate data interpretability is especially important when dashboards provide end user feedback, such as performance feedback or peer comparisons, as that feedback may result in unintended negative consequences. To improve the dashboard’s data interpretability and clarity, we recommend that dashboard developers and implementers request feedback from end users on how they interpret any dashboard communications via interviews, surveys, or Think Aloud exercises, where the end user is asked to verbalize their interpretations of the dashboard’s data [33]. Data interpretability and clarity should be assessed during the preparation and implementation phases.

#### #3: Early and ongoing multi-level needs assessment/identification of implementation barriers and facilitators (EPIS Phases: Exploration, Preparation, and Implementation)

It is not uncommon for dashboard developers and implementers to involve only limited individuals (e.g., the research team) when developing dashboards. However, by doing so, dashboards may reflect only the needs and values of those limited individuals and not the needs and values of all relevant actors (e.g., community partners). This will hinder successful implementation and use of the dashboard due to potential misalignments in goals and values. Thus, consistent with human-centered design and implementation science principles, we recommend that dashboard developers and implementers establish early and ongoing communications with relevant actors and conduct needs assessments to inform dashboard development and implementation [14, 15, 30]. Needs assessments should include multi-level perspectives from relevant actors, such as potential dashboard end users, as informed by human-centered design methods, and organizational/clinic leaders who may support dashboard implementation, as informed by implementation science methods [14]. Needs assessments for clinical or administrative dashboards, for example, may include perspectives from hospital management, clinic management, clinical providers and/or administrative staff. Relevant actors will be critical in providing key feedback about dashboard design and implementation plans. Dashboard developers and implementers should specifically seek feedback from relevant actors on how they will use the dashboard, whether the dashboard adds value or helps solve a problem, and the barriers and facilitators to dashboard implementation. Information on barriers and facilitators can help reveal factors that may hinder or support dashboard implementation and inform potential strategies to enhance implementation. We suggest using the CFIR to guide needs assessments and the identification of implementation barriers and facilitators [31, 32]. The Inventory of Factors Affecting Successful Implementation and Sustainment (IFASIS) may also be used to identify implementation barriers and facilitators [34].

#### #4: Design for health equity (EPIS Phases: Preparation and Implementation)

Consistent with the emerging health equity focus in implementation science [35], we recommend that dashboard developers and implementers carefully consider health equity when developing and implementing a dashboard. Dashboards can help enhance health equity when they promote equal access to high-quality evidence-based care for diverse populations [35]. Some dashboards, on the other hand, can hinder health equity when they display biased or inaccurate health data that exclude or misrepresent minoritized populations, as discussed above. Thus, it is imperative for dashboard developers and implementers to consider any potential data biases or gaps and how their dashboard may help perpetuate such biases. Steps should be taken to address biases, such as by acquiring more representative data, weighing data to increase the representativeness of underrepresented data segments, providing disclaimers when the dashboard is not applicable to certain populations, and/or considering alternative strategies to the dashboard that may be less biased. In all cases, especially when dashboard biases are not readily apparent, dashboard developers and implementers should acquire dashboard feedback from diverse end users and/or other groups of individuals who may be impacted by the dashboard (e.g., for clinical dashboards, patients may be impacted if end users are clinicians). Additionally, dashboard developers and implementers should assess for health equity, such as by using the Health Equity Implementation Framework (HEIF) [35] and the Digital Healthcare Equity Framework [36], during the preparation and implementation phases.

#### #5: Determine usability and selection of data visualizations (EPIS Phases: Preparation and Implementation)

For successful dashboard implementation and in line with human-centered design methods, the dashboard must be usable to end users, meaning that it is user-friendly, comprehensible, intuitive, easy to operate, and visually appealing [33]. Additionally, dashboard developers and implementers must ensure that any graphs on the dashboard are easily interpretable by end users [14, 15, 33]. Dashboards that are not usable to end users will not be used as envisioned, which can hinder implementation efforts. For example, end users may start relying on dashboard developers and implementers to interpret and communicate dashboard data rather than using the dashboard. To enhance dashboard usability, we recommend that dashboard developers and implementers collect dashboard usability data from end users, including end users’ experiences with the dashboard’s functioning, layout, and visual appearance using common human-centered design methods [33]. This data may be collected via surveys, such as the System Usability Scale (SUS) [37], and/or qualitative interviews [33]. Qualitative interviews may include Think-Aloud exercises, where the end user is asked to verbalize their thoughts and reactions as they navigate the dashboard interface [33]. Dashboard developers and implementers should collect usability information during the preparation and implementation phases. In all cases, dashboard developers and implementers can transform usability feedback into actionable recommendations for enhancing the dashboard design.

#### #6: User iterative design and evaluation (EPIS Phases: Preparation, Implementation, and Sustainment)

To enhance dashboard use, we recommend that dashboard developers and implementers engage in an iterative dashboard design and evaluation process, an important aspect of human-centered design. Specifically, it is critical for dashboard developers and implementers to gather dashboard evaluation data across diverse areas, such as dashboard usability, value, and equity. Evaluations should target end users and, if applicable, other relevant actors impacted by the dashboard. Evaluation data will help elucidate ways to enhance the dashboard and, thus, improve its effectiveness and implementation. When dashboards are not evaluated in the implementation process, it becomes unclear if and why an implementation effort may have failed. In some cases, implementation efforts fail due to dashboards not functioning as designed or end users not using the dashboard at the anticipated frequency.

We recommend that dashboard developers and implementers evaluate their dashboard multiple times during the preparation, implementation, and sustainment phases. This will allow dashboard developers and implementers to redesign or update the dashboard based on feedback and gather additional evaluation data on the redesign. This is necessary because dashboard redesigns or updates may generate new problems or expose previously hidden problems. End users may also change their perspectives on the dashboard over time due to changes in their environment or organization, such as the introduction of a new organizational policy or performance metric. It is not uncommon for dashboards to require multiple redesigns based on multiple rounds of feedback.

#### #7: Consider appropriate outcomes (EPIS Phases: Preparation, Implementation, and Sustainment)

For dashboard developers and implementers to properly evaluate their dashboard, it is critical that they select an appropriate set of outcomes (i.e., measures or estimates of how much or how well the dashboard fulfills its intended purpose), as informed by implementation science methods. The appropriate set of outcomes can often depend on the purpose of the dashboard in the implementation process. Dashboards often serve one of two purposes—the *intervention* to be implemented or the *implementation strategy* (i.e., method used to deliver the intervention). In some cases, dashboards may show characteristics of both interventions and implementation strategies and can be classified as *adjunctive interventions* [38].

When dashboards serve as the intervention, this suggests that they are directly responsible for changes in effectiveness outcomes (e.g., clinical and/or administrative outcomes). Dashboards fulfilling this purpose are viewed as necessary components to achieving the desired effectiveness outcomes, and if removed from practice, there will be significant changes to effectiveness outcomes. As an example, Laurent and colleagues (2021) developed dashboards to manage and improve the quality of anesthesiology care at a French university medical center [39]. Their dashboards focused on presenting data related to the anesthesiology unit’s overall activity, compliance with guidelines on intraoperative hemodynamics, ventilation and monitoring, and documentation of the anesthesia procedure [39].

When dashboards serve as the implementation strategy, they are the mechanism that helps support implementation of the intervention. In other words, dashboards fulfilling this purpose serve as the method, technique, or “how to” for adopting and sustaining the intervention [40]. In this role, dashboards can be characterized as a form of audit and feedback by collecting and delivering performance data to inform and change behaviors [41]. When dashboards are viewed as the implementation strategy, other implementation strategies (e.g., coaching) can be used in place of or alongside dashboards to help support implementation of the intervention. Such dashboards, in some cases, may become less useful over time and could even be removed from practice without impacting effectiveness outcomes once the intervention has been successfully implemented. An example of a dashboard serving as the implementation strategy is in Ford and colleagues’ study aimed at increasing access to MOUD in specialty addiction programs in Washington State [18, 19]. In that study, dashboards were incorporated as part of an enhanced feedback and monitoring strategy to help specialty addiction programs expand access to MOUD [18, 19]. The study compares dashboards to other implementation strategies, such as NIATx (Network for the Improvement of Addiction Treatment) external facilitation and internal facilitation, to determine which strategy or combination of strategies are most effective at increasing access to MOUD [18, 19].

When dashboards serve as the adjunctive intervention, they help recipients of the intervention adhere to or engage with the intervention [38]. Adjunctive dashboards have an indirect effect on the intervention’s primary effectiveness outcomes (e.g., clinical or administrative outcomes) and are insufficient on their own at producing these outcomes [38]. One example of a dashboard serving as the adjunctive intervention is in the Healing Communities Study, a four-year trial testing the Communities That Heal (CTH) intervention on reducing opioid overdose deaths in communities across Kentucky, Massachusetts, New York, and Ohio [7]. One component of the CTH involves engaging community members in reviewing timely opioid-related data demonstrating the extent of the problem in the community (e.g., number of overdose deaths, administration of medications to treat opioid use disorder) [7]. Community-tailored dashboards were used to support this goal of the CTH by helping community members understand and interpret opioid-related data, identify community needs, make informed decisions, and take collective action towards addressing the local opioid crisis [7].

When evaluating dashboards, we recommend that dashboard developers and implementers first discern whether their dashboard functions as the intervention, adjunctive intervention, or implementation strategy. Certain evaluation outcomes become important to assess depending on the dashboard’s purpose. Although dashboards serving as the intervention are conceptually different from those serving as the adjunctive intervention, we believe both types of dashboards require examining similar evaluation outcomes. For example, if the dashboard is the intervention or adjunctive intervention, and the intervention has not yet been shown to be effective, then it will be critical to examine effectiveness outcomes. For dashboards serving as the intervention or adjunctive intervention, it will also be critical to examine proximal and distal implementation outcomes. We recommend examining proximal implementation outcomes, such as acceptability (i.e., how agreeable is the dashboard), feasibility (i.e., extent to which the dashboard can be successfully used), and appropriateness (i.e., perceived relevance of the dashboard to address a certain problem) during the preparation phase [40]. Note that acceptability, feasibility, and appropriateness may be better classified as mediators or contributors to implementation success rather than implementation outcomes. These outcomes can be assessed using the Acceptability of Intervention Measure (AIM), Feasibility of Intervention Measure (FIM), and Intervention Appropriateness Measure (IAM) [42]. Distal implementation outcomes may be more appropriate to examine during the implementation phase, such as adoption (i.e., how many individuals use the dashboard), reach (i.e., how many individuals use the dashboard out of the number of individuals who should be using the dashboard), and use frequency (i.e., number of dashboard clicks) [43]. Dashboard sustainability (i.e., extent to which dashboard use is maintained) should be examined during the sustainability phase [43]. Adoption, reach, use frequency, and sustainability can be measured via observation [43].

Implementation outcomes can also be important to examine when the dashboard is the implementation strategy. For instance, examining dashboard acceptability, feasibility, and appropriateness during the preparation phase, and adoption during the implementation phase, can help ensure that the dashboard functions and is used as intended, thus enhancing the dashboard’s potential in supporting implementation of the target intervention. In all cases, regardless of whether the dashboard is the intervention, adjunctive intervention, or implementation strategy, we recommend that dashboard developers and implementers assess their dashboard’s usability, value, and equity to enhance the dashboard’s design and its effectiveness as an intervention, adjunctive intervention, or implementation strategy. Note that equity can be examined as an extension of various evaluation outcomes [44]. For example, dashboard developers and implementers may assess for equity when examining *reach* by determining whether all populations have access to the dashboard out of the individuals who should be using the dashboard [44]. They may also assess for equity when examining *adoption* by determining which settings and individuals adopted the dashboard [44]. See Table 1 for a summary of recommended dashboard evaluation outcomes and possible methods to assess such outcomes based on the dashboard’s purpose.

**Table 1:** Recommended dashboard evaluation outcomes based on dashboard purpose

Dashboard is the…	**Intervention or** **Adjunctive Intervention**	**Implementation Strategy**	**Recommended EPIS**^**a**^** Phase**	**Possible Assessment Methods**
**Effectiveness outcomes**	Yes, if not yet established	No^b^	Implementation	Varies depending on outcomes
**Implementation outcomes**				
**Acceptability** (how agreeable is the dashboard)	Yes	Yes	Preparation	AIM^c^
**Feasibility** (extent to which the dashboard can be successfully used)	Yes	Yes	Preparation	FIM^d^
**Appropriateness** (perceived relevance of the dashboard to address a certain problem)	Yes	Yes	Preparation	IAM^e^
**Adoption** (how many individuals use the dashboard)	Yes	Yes	Implementation	Observation
**Reach** (how many individuals use the dashboard out of the number of individuals who should be using the dashboard)	Yes	Not critical	Implementation	Observation
**Use Frequency** (number of dashboard clicks)	Yes	Not critical	Implementation	Observation
**Sustainability** (extent to which dashboard use is maintained)	Yes	No	Sustainment	Observation, CFIR^f^ interviews, IFASIS^g^
**Usability** (dashboard’s ease of use)	Yes	Yes	Preparation & Implementation	SUS^h^, Think Aloud exercises
**Value** (importance of the dashboard’s data metrics to relevant actors)	Yes	Yes	Exploration, Preparation, & Implementation	CFIR interviews
**Equity** (extent to which dashboard data are biased)	Yes	Yes	Preparation & Implementation	HEIF^i^, Digital Healthcare Equity Framework, observation

^a^EPIS is the Exploration, Preparation, Implementation, and Sustainment Framework; we list the recommended EPIS phase(s), though, outcomes may be examined in other phases, as well

^b^Except in cases where the target intervention’s effectiveness has not yet been established, such as in Hybrid Type 2 and Type 3 studies [45]

^c^Acceptability of Intervention Measure

^d^Feasibility of Intervention Measure

^e^Intervention Appropriateness Measure

^f^Consolidated Framework of Implementation Research

^g^Inventory of Factors Affecting Successful Implementation and Sustainment

^h^System Usability Scale

^i^Health Equity Implementation Framework

#### #8: Plan for sustainment (EPIS Phases: Exploration, Preparation, Implementation, and Sustainment)

Even when a dashboard is successfully implemented, this does not guarantee that its implementation and use will be sustained in the long-term. Thus, as informed by both human-centered design and implementation science methods, it is critical for dashboard developers and implementers to consider factors that will help sustain dashboard implementation and use. We recommend that dashboard developers and implementers employ three key strategies to ensure factors that will help sustain dashboard implementation and use are considered throughout each phase. First, dashboard developers and implementers should take advantage of frequent and ongoing engagement with relevant actors to learn about any potential barriers and facilitators to sustained dashboard implementation and use, such as long-term costs, existing infrastructure or platforms that may house the dashboard (e.g., the electronic health record), resources available for data transfers to the dashboards, and technical support. Barriers and facilitators to sustained dashboard implementation and use can be assessed using CFIR-informed qualitative interviews or the IFASIS [31, 32, 34].

Second, dashboard developers and implementers should utilize this information to develop solutions to barriers and maximize use of existing resources during the exploration, preparation, and implementation phases. Dashboard developers and implementers can also use this information to understand how dashboards may be integrated within existing workflows, to minimize disruptions and, thus, increase the likelihood of its sustained use [15]. For example, a dashboard that is housed within the organization’s electronic health record can more easily be integrated into existing workflows compared to a dashboard requiring access via an entirely new and different platform. Relatedly, a dashboard that depends on existing software licenses can be less costly than a dashboard that requires the organization to pay for new software licenses.

Third, dashboard developers and implementers should consider the short- and long-term needs of relevant actors to ensure that the dashboard’s value over time. Note that some dashboards may not require sustained implementation, particularly if they serve as the implementation strategy and become futile after successful implementation of the target intervention. However, we recommend that dashboard developers and implementers examine the needs of relevant actors and the barriers and facilitators to dashboard implementation, even when sustained implementation is not required. Such information can also help inform short-term dashboard implementation or use.

### Cautions

Despite the many advantages of incorporating human centered design and implementation science methods for developing effective and sustainable dashboards, we discuss some cautions of using this approach:

- #1: Human-centered design and implementation science both highlight the importance of purposefully engaging community partners and end users at every stage of the process and carefully considering their feedback on the dashboard. However, such feedback might include endless and contradictory opinions or requests for how to improve the dashboard [14]. It is necessary for dashboard developers and implementers to prioritize feedback based on importance to community partners and end users and the frequency at which the feedback is provided. Some requests may not be feasible. For example, community partners may request a dashboard feature that is not supported by the available technology. Dashboard developers and implementers should address feedback based on what is feasible. When a particular request is unfeasible, it may be necessary to inform community partners and discuss potential compromises. Additionally, in some cases, feedback on the dashboard may not align with research or operational goals. For instance, dashboard developers and implementers may be focused on developing a dashboard to enhance the quality of substance use treatments, but community partners may request that the team focus on quality enhancement of all behavioral health treatments. In such cases, it may be important for dashboard developers and implementers to discuss with community partners: 1) the intended dashboard goals, purpose, or outcome, 2) any limitations, and 3) opportunities for addressing the request in the future.

- #2: Both human-centered design and implementation science focus on meeting community partners’ and end users’ existing needs. However, this can limit opportunities for creativity. For example, dashboard developers and implementers may dismiss an idea if end users are initially reluctant to it. Dashboard developers and implementers may also refrain from considering ideas that go beyond feedback received. While it is important to consider community partners’ and end users’ needs in dashboard design and implementation, dashboard developers and implementers should also consider the potential for human adaptation to new innovations. Some innovations may be initially challenging to end users. However, end users may quickly adapt to the innovation and grow to like it over time. This is apparent in many day-to-day objects, such as smart phones, which were initially considered challenging to use and are now widely adopted. To examine dashboard adaptation trends, we recommend that dashboard developers and implementers longitudinally measure usability multiple times across the EPIS preparation and implementation phases, using the same sample of end users each time.

## Conclusions

We encourage dashboard developers and implementers to use human-centered design and implementation science methods during dashboard development and implementation to enhance dashboard use. We provide recommendations on how to incorporate human-centered design and implementation science methods to dashboard development and implementation guided by the EPIS Framework. It is possible that using this combined approach may complicate and prolong the dashboard design and implementation process. However, we believe that incorporating human-centered design and implementation science methods is critical for developing more effective, sustainable, and impactful dashboards. It is only when dashboards are effective and sustainably implemented that they can effectively promote evidence-based care practices in the community.

## Data Availability

Not applicable.

## Abbreviations

AIM: Acceptability of Intervention Measure
CFIR: Consolidated Framework of Implementation Research
CTH: Communities that Heal
EPIS: Exploration, Preparation, Implementation, and Sustainment Framework
FIM: Feasibility of Intervention Measure
HEIF: Health Equity Implementation Framework
IAM: Intervention Appropriateness Measure
IFASIS: Inventory of Factors Affecting Successful Implementation and Sustainment
MOUD: Medications for Opioid Use Disorder
NIATx: Network for the Improvement of Addiction Treatment
SUS: System Usability Scale
